# Big Five personality traits and coping strategies of Italian university students during the COVID-19 pandemic first wave

**DOI:** 10.3389/fpsyg.2023.1150674

**Published:** 2023-05-16

**Authors:** Roberto Burro, Giada Vicentini, Daniela Raccanello

**Affiliations:** Department of Human Science, University of Verona, Verona, Italy

**Keywords:** personality traits, COVID-19, coping, university students, Big Five, resilience, disaster preparation

## Abstract

**Introduction:**

Little is known about the role personality traits may have played for university students in diminishing and compensating for the negative impact of COVID-19 in its early phases, promoting adaptive coping. University students represent a population which was consistently obliged to follow social distance rules due to the early shift of many organizations from face-to-face to online learning. Therefore, it is worth exploring whether the Big Five traits acted as risk or protective factors after the outbreak of a disaster such as the COVID-19 pandemic for Italian university students.

**Methods:**

We involved a sample of 2,995 university students who completed an online survey in March 2020. We measured the Big Five personality traits through the Big Five Inventory-2-XS and their coping strategies through the Robust—Pandemic Coping Scale. The latter assessed four COVID-19-related coping dimensions, namely Despair (e.g., including helplessness and feeling lack of control), Aversion (e.g., referring to oppositive strategies), Proactivity (e.g., comprising problem solving and information seeking), and Adjustment (e.g., concerning reappraisal and assertiveness).

**Results:**

Preliminarily, two Linear Mixed Models indicated that university students had higher scores in Conscientiousness, followed by Open-Mindedness, and then Agreeableness. These three traits were, in turn, higher than Extraversion and Negative Emotionality, which did not differ among them. Concerning coping, university students reacted more frequently utilizing adaptive strategies (with Proactivity used more frequently than Adjustment) rather than maladaptive strategies (with Despair higher than Aversion). A Path Analysis examining the relations between the Big Five traits and the four coping dimensions showed that Negative Emotionality can be considered as a risk factor, and that Agreeableness, Conscientiousness, and Open-Mindedness can be conceptualized as protective factors. More interestingly, we found that Extraversion entailed both a risk and a protective role for Italian university students after the outbreak of the COVID-19 pandemic.

**Discussion:**

Notwithstanding limitations, these findings can be the basis for developing disaster preparation and prevention actions, aiming at promoting students’ positive coping towards current and future disasters.

## 1. Introduction

On 11 March, 2020, the WHO (2020) declared the COVID-19 pandemic. As all natural disasters (EM-DAT, n.d.), pandemics have potentially traumatic consequences for people of all ages and for a plethora of fields (Kumar et al., 2021). The consequences of the COVID-19 spreading have heavily affected everyday life in the social, economic, health, and education domains for millions of individuals (Kumar et al., 2021). In turn, the negative effects on physical health and the dramatic changes to which people were forced to comply with the restrictions that, worldwide, were imposed to limit the diffusion of the SARS-CoV-2 virus, have caused an increase of psychopathological symptoms and disturbances such as depression, anxiety, post-traumatic stress disorder, and others (e.g., Fitzpatrick et al., 2020; Gallagher et al., 2020; Nicomedes and Avila, 2020; Rodríguez-Rey et al., 2020; Arslan et al., 2021; Dozois and Mental Health Research Canada, 2021; García-Portilla et al., 2021). As a result of the state of emergency and the corresponding safety measures, there were a variety of disruptions in everyday routines (Ellis et al., 2020; Holmes et al., 2020). For education systems, they comprised school and university closures, with a particular emphasis on social distance, from the beginning of the outbreak of the pandemic. However, thanks to the technological and educational resources of many universities, for higher education students the interruptions of formal learning were generally very brief, given the prompt responses of many organizations to shift lessons from onsite to online learning in rapid times (Aristovnik et al., 2020). A survey which involved 424 universities and other higher education institutions based in more than 100 countries, revealed that in 2020 at least two-thirds of such organizations moved from onsite to online lessons (Marinoni and Jensen, 2020). Moreover, during 2020 there was a marked increase in the number of licenses of platforms such as Zoom (2020), which is one of the learning systems used by many universities (including the one in which this research was conducted).

In parallel with a growth of the interest in some of the psychological consequences of the outbreak of the pandemic for university students (e.g., on students’ expectations and experiences, Aucejo et al., 2020; on adjustment to online courses, Audet et al., 2021; on e-learning achievement emotions, Raccanello et al., 2022a), researchers are paying increasing attention to university students’ characteristics of personality that could have played a role in diminishing and compensating the negative impact of the diffusion of the COVID-19, promoting adaptive coping (Anglim and Horwood, 2021; Audet et al., 2021; Sahinidis and Tsaknis, 2021; Staller et al., 2021; Quigley et al., 2022; Wang et al., 2022; Weiß et al., 2022; Zolotareva et al., 2022). In line with this tendency, we explored whether the Big Five traits (i.e., Extraversion, Agreeableness, Conscientiousness, Open-Mindedness, and Negative Emotionality or Neuroticism) acted as risk or protective factors after the outbreak of a disaster such as the COVID-19 pandemic for a sample of Italian university students, taking into account a large variety of coping strategies.

In this work we focused on the Five-Factor Model (Costa and McCrae, 2006), given that this personality approach is one of the most widely used (Bacon et al., 2022). Being a universal and cross-cultural model, its use makes it easy for researchers to collaborate and compare the results from different studies; moreover, the usability of the corresponding questionnaires and scoring systems makes the model practical for a variety of professionals, and this can help particularly for dissemination of research findings (McCrae and Costa, 1997; Montag and Elhai, 2019; Marengo et al., 2021). Recent studies document also the Big Five factors’ specificity from a neurobiological perspective (DeYoung, 2015; Davis and Panksepp, 2018; Marengo et al., 2021). In the literature, there are also other models conceptualizing personality traits (e.g., Anglim and O’connor, 2019; Bacon et al., 2022). Among them, a viable alternative option to the Big Five model is offered by the HEXACO model, distinguishing Honesty-Humility, Emotionality, EXtraversion, Agreeableness, Conscientiousness, and Openness to Experience (Ashton and Lee, 2007, 2020). This model extends the Big Five model considering a sixth factor, Honesty-Humility, which corresponds to individuals’ tendency to be fair and authentic in social interactions (Bacon et al., 2022). Rather than being a mere addition, the sixth factor derives from differently portioning the variance which related to Big Five Agreeableness and Negative Emotionality to HEXACO Agreeableness, Negative Emotionality, and Honesty-Humility (Bacon et al., 2022). The HEXACO model was used to study behavioral and emotional responses in relation to risk situations, also during the pandemic (Modersitzki et al., 2020; Volk et al., 2021). However, some authors argue that this model has not been demonstrated to be universal and that the contents of the sixth factor partially overlaps with facets already included in the Big Five Agreeableness factor (Anglim and O’connor, 2019). Acknowledging the strengths and constraints of the HEXACO model, we nevertheless decided to focus on a model including the five main factors comprised by the most known models (i.e., the Big Five model), both for parsimony and for the need to rapidly assess personality traits, given the sudden and unpredictable outbreak of the pandemic (it is worth noting that the time interval between the declaration of the pandemic and the date in which we presented the project for this research to the Ethical Committee was quite short—see the Procedure section). Moreover, we needed brief instruments suitable for online administration and already available in Italian in that specific historical moment. The choice of focusing on the Big Five model was also in line with most research about personality traits and the pandemic published during 2020, as indicated by Bacon et al. (2022), who for example identified a higher number of works based on the Big Five model rather than the HEXACO model. However, the results regarding the HEXACO model tend to replicate those concerning the Big Five model (Bacon et al., 2022). Advancing research on the relation between personality traits and coping is of paramount relevance to extend our knowledge on how to support students after a disaster occurs, and also to plan in advance interventions regarding disaster preparedness and prevention. All these actions are essential to promote university students’ resilience, as the capacity to adapt positively after having experienced traumatic events (Masten, 2021).

## 2. Literature review

### 2.1. Coping strategies

The outbreak of a disease usually provokes a variety of psychological reactions (Kohút et al., 2021). The cognitive, emotional, and behavioral responses that individuals display when facing stressful events are classically defined as coping strategies (Lazarus and Folkman, 1984; Lazarus, 2000). Such strategies are activated when people perceive that the requests of an event exceed individuals’ resources for responding. Coping strategies are still relevant today to take into account the processes through which people react to stress, in particular with reference to their maladaptive or adaptive function (Ewert et al., 2021). Nevertheless, it is worth anticipating that there is a wide variety of strategies that people can use and that a key factor associated with their efficacy is the ability to flexibly choose the most adequate strategy in relation to the characteristics of both individuals and specific contexts (Ewert et al., 2021; Zimmer-Gembeck, 2021). Aiming to elaborate an extended taxonomy that could comprise most of the coping strategies studied within the psychological literature, Skinner and Zimmer-Gembeck (Zimmer-Gembeck and Skinner, 2011; Skinner and Zimmer-Gembeck, 2016; Zimmer-Gembeck, 2021—for adaptation of this categorization to natural disasters, see Raccanello et al., 2020, 2021, 2023; to the pandemic, see Burro et al., 2021; to wars, see Vicentini et al., 2022) proposed a classification based on the concept that an uncertain or stressful event can be perceived as a threat or as a challenge towards the three basic human needs for competence, relatedness, and autonomy (Deci and Ryan, 1985). These three needs are at the core of the Self-Determination Theory, according to which individuals’ wellbeing and psychological functioning is associated with the satisfaction (and the absence of frustration) of such needs (Deci and Ryan, 1985). The need for autonomy concerns the human tendency towards autonomous choices and critical thinking; the need for competence regards people’s need for mastering the contextual requests and perceiving oneself as competent; and the need for relatedness refers to the tendency of experiencing positive relations in which people care one another (Šakan et al., 2020). Coherently with the Self-Determination Theory, recent findings suggested that, also during the pandemic, satisfaction and frustration of the three basic psychological needs is relevant for influencing individuals’ wellbeing (Šakan et al., 2020). This has been investigated also involving university students during the first wave of the pandemic, for example indicating that Conscientiousness is related to low levels of frustration and high levels of satisfaction of the competence need (Staller et al., 2021).

When an event is perceived as a challenge, it is likely that the individuals’ responses would be adaptive; when it is perceived as a threat, the probability of them being maladaptive would raise. For each of the six intersections between these two aspects (threat vs. challenge perception; type of need) there would be four families of coping strategies, two maladaptive and two adaptive. For example, at the outbreak of the pandemic people lacked knowledge on how to face it, both because authorities needed some time to implement the first rules to limit the diffusion of the virus, and because vaccines were not available yet. According to how individuals perceived this as a threat or a challenge to their competence, respectively, they could have reacted in maladaptive ways, e.g., thinking that no solution was possible or trying to cognitively escape from the reality denying it, or in adaptive ways, e.g., trying to behave according to the prevention guidelines or to search for information. When a stressful event regards the need for:

- Competence, the two maladaptive reactions can be classified within the families, respectively, of *helplessness* and *escape*, while the two adaptive reactions within the families of *problem solving* and *information seeking*.
- Relatedness, the maladaptive families include *delegation* (e.g., feeling of being out of control) and *social isolation* (e.g., withdrawing from social interactions), and the adaptive families *self-reliance* (e.g., focusing on emotion awareness and regulation) and *support seeking* (e.g., giving or looking for social support).
- Autonomy, the maladaptive families comprise *submission* (e.g., ruminating) and *opposition* (e.g., refusing to cooperate), and the adaptive families *accommodation* (e.g., using reappraisal) and *negotiation* (e.g., being assertive and contracting, identifying priorities).

Based on this taxonomy, we developed a 20-item scale to assess maladaptive and adaptive coping strategies related to pandemics using the Rasch modeling, i.e., the Robust—Pandemic Coping Scale (R-PCS; Burro et al., 2021). Through a dual approach combining an exploratory and a confirmatory analysis, we identified the four dimensions of the scale, called Despair, Aversion, Proactivity, and Adjustment. The first two dimensions focused on threats while the other two on challenges. Based on previous literature (Zimmer-Gembeck and Skinner, 2011; Skinner and Zimmer-Gembeck, 2016) and on their relations with positive (i.e., enjoyment) and negative (i.e., anger) emotions, we could argue that the first two are maladaptive while the other two are adaptive. To help clarity, we report in Table 1 the items of the four dimensions of the R-PCS, distinguishing them according to their reference to threats or challenges; to the needs for competence, relatedness, or autonomy; and to the family of coping strategies (helplessness, delegation, submission, opposition, problem solving, information seeking/giving, social support, accommodation, and negotiation). In particular:

- *Despair* included items referred to threats towards the three needs, i.e., competence, relatedness, and autonomy; they concerned the families of helplessness, delegation, and submission.
- *Aversion* comprised only items about threats to autonomy, and specifically pertaining to the family of opposition.
- *Proactivity* included items related to challenges towards competence, about problem solving and information seeking/giving, and only one item about relatedness (which was however focused on an active way to solve problems).
- *Adjustment* included items pertaining to challenges towards autonomy, concerning accommodation and negotiation, and one item about relatedness (focused on relations but referred also to contracting with others).

**Table 1:** Items of the four dimensions of the R-PCS (in italics), distinguished according to their reference to threats vs. challenges; competence, relatedness, or autonomy needs; and family of coping strategies (helplessness, delegation, submission, opposition, problem solving, information seeking/giving, social support, accommodation, and negotiation).

	Pandemic perceived as a threat		Pandemic perceived as a challenge	
R-PCS Dimension	Despair	Aversion	Proactivity	Adjustment
Need for competence	Helplessness: *Thinking that nobody can help me*		Problem solving: *Behaving in safe ways (for example washing my hands frequently)*		Information seeking/giving: *Giving correct, clear, and comprehensible information* *Looking for information from reliable sources* *In case of doubts, asking for information on appropriate behaviors*
			Need for relatedness		Delegation: *Panicking*		Social support: *Helping and reassuring those around me*	Social support: *Collaborating with others*
	Need for autonomy				Submission: *Overthinking about the emergency* *Thinking that things will never get better*		Opposition: *Remembering that following the rules protects everybody’s health* (reversed) *Thinking that safety measures are not useful* *Following advice from experts* (reversed) *Ignoring the regulations from the Ministry of Health*		Accommodation: *Taking the opportunity to cultivate hobbies* *Keeping myself busy (for example playing or studying)*
			Negotiation: *Creating new routines if usual ones cannot be followed* *Focusing on things that are really important (for example family)*					

### 2.2. Personality traits

Students’ cognitive, emotional, and behavioral reactions at the outbreak of the COVID-19 pandemic may have been associated with different risk or protective factors. Risk factors have a negative impact on psychological development and functioning, worsening them, while protective factors are those contributing to their amelioration (Luthar et al., 2015). Could personality traits have played a role as risk or protective factors in such period for Italian university students’ coping?

The Big Five personality trait domains enabled the organization of individual differences according to typical patterns of thoughts, feelings, and behaviors (Soto and John, 2017). Some conceptualizations distinguish also specific facet-traits within each larger Big Five domain, with a hierarchical organization (McCrae and Costa, 2010; Soto and John, 2017). Within the so-called “person-situation debate” (Goldberg, 1993; Faulkner et al., 2004; Fink et al., 2021), personality traits have generally been recognized as stable within individuals and consistent across time and situations (Fink et al., 2021). However, data on adults seem to suggest that in the case of the pandemic the way in which individuals perceived the situation explained more variance related to adaptive reactions such as compliance with safety behaviors than their personality, for which it was usually quite low (Zajenkowski et al., 2020; Fink et al., 2021). Nevertheless, not much is known on the relation between personality traits and a wide range of pandemic-related coping strategies for the specific population of university students, which was obliged to abruptly diminish face-to-face social interactions due to the shift to online lessons at the COVID-19 outbreak (see paragraph 2.4. Big Five Traits and Coping Strategies in University Students During the Pandemic below for studies on this issue).

It is worth noting that personality traits could have different effects in the various phases of the disaster cycle (i.e., nondisaster phase, predisaster phase, impact phase, emergency phase, and reconstruction phase; Noji, 1997; other researchers refer to similar and partially overlapping phases such as mitigation, preparedness, response, and recovery phases; Alexander, 2019; Sawalha, 2020). We focused on the impact phase, corresponding to the moment of the occurrence of the disaster which can provoke serious damages for things and people. The relatively long duration of the impact phase for the COVID-19 pandemic gave the rare possibility to study the role of personality in a time frame which is generally under-investigated, due to its very short duration for many natural disasters (e.g., earthquakes, tsunamis, or volcanic eruptions).

On the basis of the literature about the Big Five, we generally expect Extraversion, Agreeableness, Conscientiousness, and Open-Mindedness to act as protective factors, and Negative Emotionality (or Neuroticism) to act as risk factors in a variety of situations.

- *Extraversion.* Considering the facet-traits distinction (McCrae and Costa, 2010; Soto and John, 2017), extraverted people can be described as sociable, assertive, and full of energy. They are usually expected to be actively engaged in social relations (McAdams, 2015). They cope with events through reappraisal (Balzarotti et al., 2010) and negotiate with others using an assertive rather than a passive or an aggressive style (Bagherian and Mojambari, 2016; Sims, 2017). Moreover, this trait is frequently associated with subjective wellbeing (Li et al., 2015; Anglim et al., 2020).
- *Agreeableness.* Agreeable people are characterized by compassion, are respectful, and think the best about other individuals. They are emphatic, care about others, and are prone to prosocial behaviors such as helping or donating (Habashi et al., 2016; Sims, 2017).
- *Conscientiousness.* Conscientious people are organized, persistent when engaged in a task, and reliable. They are used to respect rules and recommendations, positively valuing achievement, order, hard work, and efficiency (Barrick and Mount, 1991; Roberts et al., 2005), and also utilizing, especially when goal progress is threatened, an assertive style (Bagherian and Mojambari, 2016; Sims, 2017). They also tend to avoid germs (Costa and McCrae, 2006). Moreover, this trait is particularly relevant for school performance (Judge and Ilies, 2002).
- *Open-Mindedness.* People with high Open-Mindedness are fascinated by art, intellectually curious, and creative. Thus, they are open to new experiences and capable of adapting easily to them (Schmutte and Ryff, 1997; McAdams, 2015), also in relation to shifting to online learning (LePine et al., 2000). In addition, they are prone to prosocial behavior (Kline et al., 2019).
- *Negative Emotionality.* People with high Negative Emotionality are anxious, depressed, and easily change their mood. They are generally characterized by poor wellbeing, psychological distress, and high scores for indicators of negative psychological functioning (Anglim et al., 2020). Low Negative Emotionality, together with high Conscientiousness, has the strongest associations with both physical and mental health (Bogg and Roberts, 2013; Friedman and Kern, 2014; Strickhouser et al., 2017). Moreover, neurotic people tend to react through maladaptive coping (Costa and McCrae, 2006), rarely using reappraisal (Balzarotti et al., 2010). They are also seldom assertive (Bagherian and Mojambari, 2016; Sims, 2017).

Finally, Agreebleness, Conscientiousness, and Open-Mindedness are usually also associated with subjective wellbeing—even if less strongly compared to the other two traits—while the relations with negative indicators of psychological functioning are usually less consistent (Anglim et al., 2020).

### 2.3. Big Five traits and COVID-19 coping strategies

Which COVID-19 coping dimensions (conceptualized according to the R-PCS) could we expect in university students on the basis of the literature about Big Five factors and coping? Does pandemic-related research support previous findings about the risk and protective role of the Big Five traits? During the pandemic, most of the research on personality and coping focused on adults spanning from 18  years to older ages, and only rarely focused on university students (see paragraph 2.4. Big Five Traits and Coping Strategies in University Students During the Pandemic below for studies on this issue). It is also worth noting that, in general, the feeling of lack of control over the unknown threats due to the outbreak of the pandemic is generally associated with misinformation and conspiracy theories (Šrol et al., 2021), in turn increasing the intensity of the perceived danger and the probability to activate personality expressions (Bedford-Petersen and Saucier, 2021). We anticipate that most of the studies which are commented on within this paragraph were conducted with adults (and not specifically with university students) during the first wave of the pandemic (i.e., Bogg and Milad, 2020; Carvalho et al., 2020; Chan et al., 2020; Muto et al., 2020; Nofal et al., 2020; Qian and Yahara, 2020; Zajenkowski et al., 2020; Aschwanden et al., 2021; Blagov, 2021; Fink et al., 2021; Götz et al., 2021; Kocjan et al., 2021; Kohút et al., 2021; Nikčević et al., 2021; Schmiedeberg and Thönnissen, 2021; Zettler et al., 2021; Abdelrahman, 2022).

#### 2.3.1. Despair

While no previous study examined the Big Five traits in relation to COVID-19 Despair as conceptualized by the R-PCS, we could gather some hints about possible links examining the COVID-19 literature assessing adults’ emotional responses, wellbeing, stress, anxiety, depression, and other symptoms. These constructs are included in the three coping families within Despair, i.e., helplessness, implying the absence of hope about the future; delegation, typical of people overwhelmed by negative emotions; and submission, manifesting itself primarily through rumination. On the whole, the COVID-19-related literature reports that various indicators of negative distress are related positively with Extraversion and Negative Emotionality, and negatively with Agreeableness, Conscientiousness, and Open-Mindedness (studies documenting these findings are detailed as follows).

Data on adults indicated that Extraversion had a positive effect on subjective wellbeing (Kocjan et al., 2021) and it was negatively correlated with generalized anxiety and depressive symptoms (Nikčević et al., 2021), but it was also positively associated with stress (Kocjan et al., 2021), negative emotional responses (Kohút et al., 2021), and a more negative perception of the situation, but only for participants without a partner (Schmiedeberg and Thönnissen, 2021). A review on the role of personality in COVID-19-related emotions and behaviors indicated that Extraversion was associated with people’s reluctance to socially distancing from others (Bacon et al., 2022). Agreeableness and Conscientiousness had a positive effect on subjective wellbeing (Kocjan et al., 2021) and were negatively correlated with generalized anxiety and depressive symptoms (Nikčević et al., 2021). Negative Emotionality was associated negatively with subjective wellbeing (Kocjan et al., 2021) and positively with negative emotions (Kohút et al., 2021), with a more negative perception of the restrictions to daily life (Schmiedeberg and Thönnissen, 2021), and with stress, anxiety, and depression (Qian and Yahara, 2020; Kocjan et al., 2021; Nikčević et al., 2021). Some studies also reveal that, among the five traits, Negative Emotionality was the strongest predictor of the worst psychological functioning (Kocjan et al., 2021) and was most related with a poor mental health (Bacon et al., 2022). Open-Mindedness was negatively linked with generalized anxiety and depressive symptoms (Nikčević et al., 2021), but it also had negative effects on subjective wellbeing (specifically, when mediated by individuals’ resilience) and positive effects on stress (Kocjan et al., 2021).

#### 2.3.2. Aversion

If we focus on the oppositive coping strategies characterizing Aversion, it is useful to take into account research about adults’ compliance with preventive measures during the COVID-19 pandemic. Considering samples of adults from a variety of countries, the current findings seem to suggest that, on the one hand, adherence to safety behaviors is higher for people with high Agreeableness, Conscientiousness, Negative Emotionality, and Open-Mindedness. On the other hand, it could be particularly difficult for extraverted individuals, especially for rules imposing social distancing.

Fink et al. (2021) found that scores in the scale Adherent Safety Behavior negatively correlated with Extraversion, while the other four traits were positively related, with the highest association for Negative Emotionality. Other findings indicated that preventive behavior was associated positively with Agreeableness (Chan et al., 2020; Muto et al., 2020; Qian and Yahara, 2020; Zajenkowski et al., 2020; Krupić et al., 2021; Pilch et al., 2021), Conscientiousness (Carvalho et al., 2020; Chan et al., 2020; Nofal et al., 2020; Qian and Yahara, 2020; Krupić et al., 2021; Abdelrahman, 2022), Negative Emotionality (Chan et al., 2020), and Open-Mindedness (Qian and Yahara, 2020; Pilch et al., 2021), and negatively with Extraversion (Krupić et al., 2021). Similar results were reported by Peters et al. (2023), commenting that, albeit with some exceptions, individual differences in the Big Five traits are linked with a range of COVID-19-related behaviors, like social distancing (Bogg and Milad, 2020; Aschwanden et al., 2021; Zettler et al., 2021), compliance with hygiene rules (Blagov, 2021; Zettler et al., 2021), and adherence to lockdown restrictions (Zajenkowski et al., 2020; Götz et al., 2021; Siritzky et al., 2022). However, the results are not always consistent. Some studies found no significant relations between the Big Five traits and compliance with recommendations (Kohút et al., 2021), or even negative relations between Negative Emotionality and adherence to recommendations (Bogg and Milad, 2020).

#### 2.3.3. Proactivity

Considering information seeking (concerning the competence need)—which is one of the most representative families measured by Proactivity—research on COVID-19 found that in the first wave Negative Emotionality positively predicted search for information (Kohút et al., 2021). Given that Proactivity included also one item about problem solving, the same literature considered in relation to compliance with rules could be taken into account for formulating the hypotheses about the relation between the Big Five traits and this coping dimension, with the opposite direction.

#### 2.3.4. Adjustment

Concerning Adjustment, Kohút et al. (2021) found that Conscientiousness negatively predicted relaxation and emotional improvement, a strategy similar to reappraisal (which is one of the coping strategies comprised by the family of accommodation), which however was positively predicted by Open-Mindedness. Schmiedeberg and Thönnissen (2021) found that Open-Mindedness was associated with a more positive perception of the situation, again an aspect that could be linked with reappraisal. As regards social support, Kohút et al. (2021) documented that Agreeableness predicted helping others.

### 2.4. Big Five traits and coping strategies in university students during the pandemic

University students constitute a population with some different characteristics from the adult population. In the period following the outbreak of the pandemic, they were generally deprived uniformly of their social interactions, in particular of those concerning learning-teaching contexts, including both peers and teachers (Aristovnik et al., 2020; Marinoni and Jensen, 2020). Using a sort of quasi-experimental design, involving university students during the pandemic in studies about personality traits and coping strategies, gave the rare possibility to focus on the effects of social deprivation on the relation between the two constructs.

Previous research involving university students has already in part examined the role of the Big Five traits concerning coping (i.e., Anglim and Horwood, 2021; Audet et al., 2021; Sahinidis and Tsaknis, 2021; Staller et al., 2021; Quigley et al., 2022; Wang et al., 2022; Weiß et al., 2022; Zolotareva et al., 2022).

For example, Anglim and Horwood (2021) involved a sample of more than 1,000 Australian undergraduates during July 2020. Their data indicate substantial similarities in the relation between the Big Five factors and wellbeing with the pre-pandemic phases, but they also revealed a reduced impact of Extraversion on positive affect, supporting the relevance of situation selection beyond temperament for explaining the impact of personality traits. Audet et al. (2021) indicated that Open-Mindedness was positively associated with subjective wellbeing and engagement towards online learning for 350 Canadian university students in the 2020 fall semester. Involving younger students (i.e., a sample of 347 Flemish secondary school students) in June 2020, Iterbeke and De Witte (2022) also found that more conscientious and open-minded students adjusted well to the changes induced by the pandemic, while more neurotic students showed higher levels of stress. In line with this, Quigley et al. (2022) reported positive correlations higher than 0.35 between Neuroticism and stress for a sample of about 300 first-year English undergraduate students in a survey from January to March 2021. Sahinidis and Tsaknis (2021) involved more than 500 Greek university students from March to April 2020, indicating that engagement with online learning was higher for more open-minded and conscientious students and lower for neurotic ones. Also vitality, regarding energy and enthusiasm, related positively to Conscientiousness and negatively to Neuroticism, in a sample of German bachelor/master students participating to an online survey in June 2020 (Staller et al., 2021). A study by Wang et al. (2022) indicated that perceived stress was related negatively with Extraversion, Agreeableness, Conscientiousness, and Open-Mindedness, and positively with Neuroticism, and there was an opposite pattern for social support. This was revealed involving a sample of almost 600 Chinese medical university students in November 2021. Using a small sample (*N* = 51), Weiß et al. (2022) focused on how the Big Five personality traits related to coping with contact restrictions in May 2020 in Germany. Their findings confirmed the association between being extravert on the one hand, and suffering from constraints together with benefiting from their relaxation on the other hand. Finally, Zolotareva et al. (2022) investigated the relation between the Big Five traits and healthy life style in March and April 2021 in a sample of more than 1,200 university students in Russia. They found, for example, that stress management was related positively with Extraversion and Conscientiousness, and negatively with Neuroticism. Moreover, social support for healthy practices was positively related with Extraversion, Agreeableness, and Conscientiousness.

Overall, most of these studies focused on constructs that are related to the R-PCS Despair factor. However, the specific characteristics of the relation between the five factors and a wide range of coping strategies as those assessed through instruments such as the R-PCS has not been examined thoroughly yet. In addition, involving university students in the very first phases of the pandemic (in our case, the participants were Italian university students), we could investigate this relation in a phase of disasters, i.e., the impact phase (Noji, 1997; Alexander, 2019; Sawalha, 2020), which is generally under-investigated given its usual short duration. For example, for other natural disasters such as earthquakes or tsunamis it is both procedurally and ethically very difficult to involve people as study participants during such phase. Therefore, knowledge about the individuals’ psychological reactions during the impact phase is frequently studied through retrospective reports (Raccanello et al., 2022b), which however can be biased by memory distortion processes.

To sum up, the recent literature about how adults, and in particular university students, coped with the threats and challenges of the pandemic in its early stages seems to give support to the well-established protective role of Agreeableness, Conscientiousness, and Open-Mindedness, and to the risk role of Negative Emotionality. Nevertheless, a great exception is played by Extraversion, whose typically protective role has been deeply questioned by the restrictions about social distancing. Whether this pattern also characterizes Italian university students in relation to a large variety of coping strategies has not been deeply investigated yet.

## 3. Aims of the current study

The current study aims at broadening the understanding of personality traits as key risk or protective factors in relation to how Italian university students coped with the challenges due to the outbreak of the COVID-19 pandemic in 2020. There are at least three gaps in the current literature that our research identified on the relations between the Big Five traits and coping with this population. To our knowledge, scarce attention has been paid at studying: (a) the Big Five traits in Italian university students during the pandemic; (b) the correlates of the Big Five traits regarding a wide range of coping strategies in Italian university students during the first wave of COVID-19; so, before generalizing suggestions concerning broader samples of adults and other countries’ university students, it is necessary to test whether the underlying theoretical assumptions are confirmed with them; and (c) risk and protective factors playing a role in the very first period of a disaster such as a pandemic (using the first wave of the COVID-19 pandemic as an example), i.e., its impact phase, as a necessary step in implementing actions to support university students’ resilience before, during, and after disasters.

Therefore, we had two main aims. The first aim was to explore how a sample of Italian university students was characterized in terms of the Big Five personality traits and COVID-19-related coping strategies assessed after the outbreak of the COVID-19 pandemic. The strategies were evaluated through the R-PCS, which measures four dimensions, namely Despair, Aversion, Proactivity, and Adjustment. The second aim was to test whether and how the Big Five personality traits were linked to the four types of COVID-19-related coping strategies. Based on previous research, we formulated four hypotheses.

*Hypothesis 1*: For Despair, we hypothesized positive links with Extraversion and Negative Emotionality, and negative links with Agreeableness, Conscientiousness, and Open-Mindedness.

*Hypothesis 2*: For Aversion, we hypothesized a positive link with Extraversion, and negative links with Agreeableness, Conscientiousness, Negative Emotionality, and Open-Mindedness.

*Hypothesis 3*: For Proactivity, we hypothesized positive links with Extraversion, Agreeableness, Conscientiousness, and Open-Mindedness, and a negative link with Negative Emotionality.

*Hypothesis 4*: For Adjustment, we hypothesized positive links with Extraversion, Agreeableness, Conscientiousness, and Open-Mindedness, and a negative link with Negative Emotionality.

To sum up, for Italian university students after the outbreak of the COVID-19 pandemic, we expected Negative Emotionality to act as a risk factor, and Agreeableness, Conscientiousness, and Open-Mindedness to act as protective factors. However, the question about the role of Extraversion remained unclear.

## 4. Method

### 4.1. Participants

We involved a sample of 2,995 participants (*M_age_* = 25.50 years, SD = 6.61, 80% females) from a North-Eastern Italian university. Respectively, the 57.45% of them were bachelor’s students, the 36.78% master’s students, and the 5.77% attended a PhD or other post-lauream courses. As regards their area of study, they were studying human sciences (25.73%), medicine (18.16%), foreign languages (17.00%), economy (14.27%), science and engineering (8.36%), literature (7.58%), law (5.43%), or sport science (3.48%). At the time of the survey, 0.37% of participants had directly experienced COVID-19, having received a positive test result, 1.46% had been tested but had a negative result, and 98.17% had never been tested.

### 4.2. Procedure

This study is part of a larger longitudinal project that involved all the students from a North-Eastern Italian university from March 2020 to July 2022 with a total of 14 waves (Burro et al., 2021). It was approved by the Director of the Head Office General Management and by the Ethical Committee of the Department of Human Sciences of the same university (protocol n. 118,846/2020; the request was presented on 13 March 2020 and it was approved on 16 March 2020). We contacted about 25,000 students by email for the first time between March and April 2020. They were invited to take part to an online survey about COVID-19, personality, and coping strategies. Students that confirmed their participation had to sign an informed consent before filling in the questionnaire. All the questions were presented in Italian. The data considered in this study concern the first wave. We administered the questionnaire about 2 weeks after that the WHO had declared the COVID-19 pandemic (11 March 2020, World Health Organization, 2020), specifically between 23 March 2020 and 1 April 2020. In Italy, on 9 March a Decree of the President of the Council of Ministers (Decreto del Presidente del Consiglio dei Ministri, DPCM; Gazzetta Ufficiale della Repubblica Italiana, 2020) had already been applied to the whole country, containing strict regulations to mitigate the diffusion of the virus and limiting, in particular, people’s mobility. At the university involved for this study, lessons shifted to completely online from the beginning of March 2020.

#### 4.2.1. Measures

##### 4.2.1.1. Big Five

For measuring the Big Five personality traits we used the Italian version of the Big Five Inventory-2-XS (BFI-2-XS; Soto and John, 2017). This instrument consists of 15 items to be rated on a five-point scale (1 = *not at all* and 5 = *very much*) according to how true each sentence is to describe the participant. It comprises five factors corresponding to the Big Five traits: Extraversion (e.g., *I am full of energy*; *ω* = 0.65), Agreeableness (e.g., *I assume the best about people*; *ω* = 0.63), Conscientiousness (e.g., *I am reliable, others can always count on me*; *ω* = 0.60), Negative Emotionality (e.g., *I worry a lot*; *ω* = 0.69), and Open-Mindedness (e.g., *I am original, I come up with new ideas*; *ω* = 0.62). Each dimension includes three items, one for each facet of the personality traits: sociability, assertiveness, and energy level for Extraversion; compassion, respectfulness, and trust for Agreeableness; organization, productiveness, and responsibility for Conscientiousness; anxiety, depression, and emotional volatility for Negative Emotionality; aesthetic sensitivity, intellectual curiosity, and creative imagination for Open-Mindedness.

##### 4.2.1.2. Pandemic-related coping strategies

We measured the strategies used to cope with the pandemic using the R-PCS (Burro et al., 2021). It comprises 20 items to be rated on a five-point scale (1 = *never* and 5 = *always*) according to how frequent each strategy is used. We asked to the participants to respond to thinking about the ways they had used to cope with emotions such as fear, sadness, and anger since the restriction measures were applied to the whole Italian territory (DPCM of 9 March 2020). The R-PCS evaluates two maladaptive, i.e., Despair (*ω* = 0.75) and Aversion (*ω* = 0.61), and two adaptive dimensions, i.e., Adjustment (*ω* = 0.68) and Proactivity (*ω* = 0.68), each including five items. The dimensions differ for how they refer to threats or challenges, and to competence, relatedness, and/or autonomy needs (Zimmer-Gembeck and Skinner, 2011; Skinner and Zimmer-Gembeck, 2016; Zimmer-Gembeck, 2021). Despair comprises negative strategies reflecting immobility and panicking reactions, with one item focused on threats towards competence (e.g., *Thinking that nobody can help me*), two towards relatedness (e.g., *Panicking*), and two towards autonomy (e.g., *Thinking that things will never get better*). Aversion regards the opposition to those rules and constraints developed by authorities to protect physical health, with five items focused on autonomy-related threats (e.g., *Ignoring the regulations from the Ministry of Health*). Adjustment concerns the positive and constructive ways that people use to face stressful situations, with one item focused on challenges towards relatedness (e.g., *Collaborating with others*) and four items towards autonomy (e.g., *Creating new routines if usual ones cannot be followed*). Proactivity refers to the efforts for finding solutions to problems, with five items focused on competence-related challenges (e.g., *Looking for information from reliable sources*). For each dimension, we transformed the scores into a Rasch-logit interval scale (ranging from 1 to 10), in line with the original scale (Burro et al., 2021).

### 4.3. Data analysis

We analyzed the data with the R software, version 4.2.2 (R Core Team, 2022), given its characteristics such as versatility, effectiveness of data visualization, community support, reproducibility, integration with other tools, and open-source characteristics (Kuhn and Johnson, 2019; Baumer et al., 2020; Peng and Matsui, 2021). We ran two Linear Mixed Models (LMM, using primarily lme4 R-package, version 1.1.31, Bates et al., 2015) and a Path Analysis (PA, using primarily lavaan R-package, version 0.6–13, Rosseel, 2012; Oberski, 2014; for applications see Raccanello et al., 2022a).

Preliminarily, we examined the descriptive statistics of all the variables that we assessed (see Table 2 for descriptive statistics and intercorrelations). We consider correlations between 0.10 and 0.30 as small, between 0.30 and 0.50 as moderate, and higher than 0.50 as large (Cohen, 1988; Baguley, 2009; Hayes, 2020). Then, we investigated whether the five factors of the BFI-2-XS differed among them through a first LMM, with participants as the random effect and factors of the scale as the categorical fixed effect; the scores of each factor were the dependent variables. We ran another LMM to study possible differences between the dimensions of the R-PCS, with participants as the random effect and dimensions of the R-PCS as the categorical fixed effect; the scores of each dimension of the R-PCS were the dependent variables. For each LMM, we performed a type III analysis of variance table with Satterthwaite’s method (Baguley, 2012; Andreasen and McDonald, 2021). We used the Bonferroni correction for post-hoc tests (using primarily emmeans R-package, version 1.8.3, Lenth, 2022). The level of significance was *p* < 0.05. Finally, we conducted a PA (Hoyle, 2012; Schumacker and Lomax, 2016) in order to examine the relations between the variables based on the theoretical assumptions derived by the literature. PA can be used to test the validity of a model and to estimate the strength of the relations between the variables. Path modeling is currently a standard approach for representing and studying direct and indirect effects of one or more independent variables on one or more dependent variables in the social sciences (Cook and Forzani, 2020). In our case, we tested a model in which the five factors of the BFI-2-XS related to the four dimensions of the R-PCS. We utilized the SEM function in the lavaan package, with the Maximum Likelihood with Robust Huber-White standard errors and scaled test statistic (MLR). For running a PA, the minimum ratio between number of observations and number of parameters should be 5:1 or more, and preferably 10:1 (Kline, 2016). In our case the ratio was 100:1, and therefore the sample size was adequate.

**Table 2:** Intercorrelations and descriptive statistics (means, *M*; standard deviations, *SD*; 95% confidence intervals, CI) for the five factors of the BFI-2-XS and the four dimensions of the R-PCS.

Variable	1	2	3	4	5	6	7	8	9
1. BFI-2-XS—extraversion	-								
2. BFI-2-XS—agreeableness	−0.05**	-							
3. BFI-2-XS—conscientiousness	0.26***	0.16***	-						
4. BFI-2-XS—negative emotionality	0.06**	−0.06***	−0.27***	-					
5. BFI-2-XS—open-mindedness	0.19***	0.20***	0.11***	−0.02	-				
6. R-PCS—despair	0.14***	−0.07***	−0.11***	0.45***	−0.08***	-			
7. R-PCS—aversion	−0.01	−0.14***	−0.13***	0.02	−0.10***	0.11***	-		
8. R-PCS—proactivity	0.15***	0.18***	0.25***	−0.11***	0.23***	−0.15***	−0.31***	-	
9. R-PCS—adjustment	0.17***	0.24***	0.25***	−0.16***	0.30***	−0.22***	−0.19***	0.45***	-
*M*	2.95	3.50	3.73	2.92	3.57	3.13	2.84	6.84	6.47
*SD*	0.57	0.69	0.71	0.85	0.74	1.17	1.20	1.47	1.20
95% CI	[2.92, 2.97]	[3.48, 3.53]	[3.71, 3.76]	[2.89, 2.95]	[3.55, 3.60]	[3.09, 3.17]	[2.79, 2.88]	[6.79, 6.89]	[6.42, 6.51]

*p < 0.05, **p < 0.01, ***p < 0.001.

## 5. Results

### 5.1. Preliminary analyses

The descriptive statistics and the intercorrelations concerning the five factors of the BFI-2-XS and the four dimensions of the R-PCS are shown in Table 2. Examining the intercorrelations between the Big Five factors, we found that both Extraversion and Agreeableness had small positive correlations with Conscientiousness and Open-Mindedness, respectively. Moreover, Conscientiousness was linked to Open-Mindedness through a small positive correlation and to Negative Emotionality through a moderate negative correlation. Concerning the R-PCS, we found positive correlations, respectively, between the two maladaptive (i.e., Despair and Aversion) and the two adaptive (i.e., Adjustment and Proactivity) dimensions—the first small and the second moderate. In addition, all the correlations between a maladaptive and an adaptive dimension were small and negative, except the one between Aversion and Proactivity, which was moderate and negative.

### 5.2. Linear mixed models (Aim 1)

**Figure 1:**
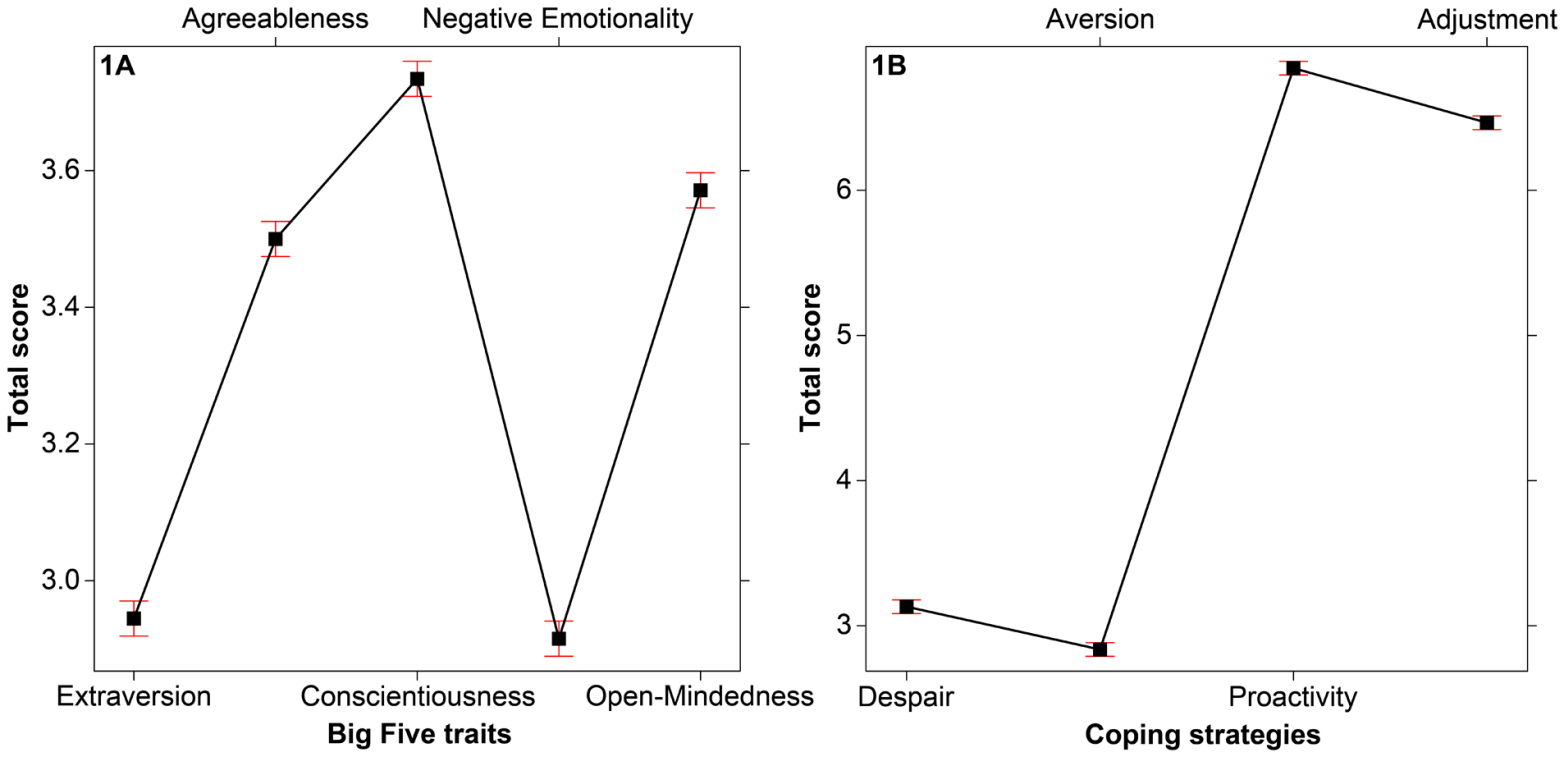
Total scores of **(A)** the five factors of the BFI-2-XS, and of **(B)** the four dimensions of the R-PCS. The bars represent the 95% CI.

The first LMM revealed a significant effect of the Big Five factors, *F*(4, 11,980) = 868.21, *p* < 0.001, *η^2^_p_* = 0.22 (Figure 1A). The post-hoc tests indicated that the scores were higher for Conscientiousness (*M* = 3.73, *SD *= 0.71, 95% CI [3.71, 3.76]) compared to Open-Mindedness (*M* = 3.57, *SD *= 0.74, 95% CI [3.55, 3.60]; *t* = 8.99, *p* < 0.001), higher than Agreeableness *M* = 3.50, *SD* = 0.69, 95% CI [3.48, 3.53]; *t* = 12.92, *p* < 0.001). In turn, Agreeableness was higher than Extraversion (*M* = 2.95, *SD* = 0.57, 95% CI [2.92, 2.97]; *t* = 30.62, *p* < 0.001 and Negative Emotionality *M* = 2.92, *SD* = 0.85, 95% CI [2.89, 2.95]; *t* = 32.25, *p* < 0.001), which did not differ among them.

The second LMM indicated a significant effect of the R-PCS dimensions, *F*(3, 11,980) = 7878.70, *p* < 0.001, *η^2^_p_* = 0.66 (Figure 1B). The post-hoc tests revealed higher scores for Proactivity (*M* = 6.84, *SD* = 1.47, 95% CI [6.79, 6.89]) compared to Adjustment (*M* = 6.47, *SD* = 1.38, 95% CI [6.42, 6.51]; *t*  = 11.10, *p* < 0.001), higher versus Despair (*M* = 3.13, *SD* = 1.17, 95% CI [3.09, 3.17]; *t*  = 98.36, *p* < 0.001), in turn higher than Aversion (*M* = 2.83, *SD* = 1.20, 95% CI [2.79, 2.88]; *t* = 8.70, *p* < 0.001).

### 5.3. Path analysis (Aim 2)

**Figure 2:**
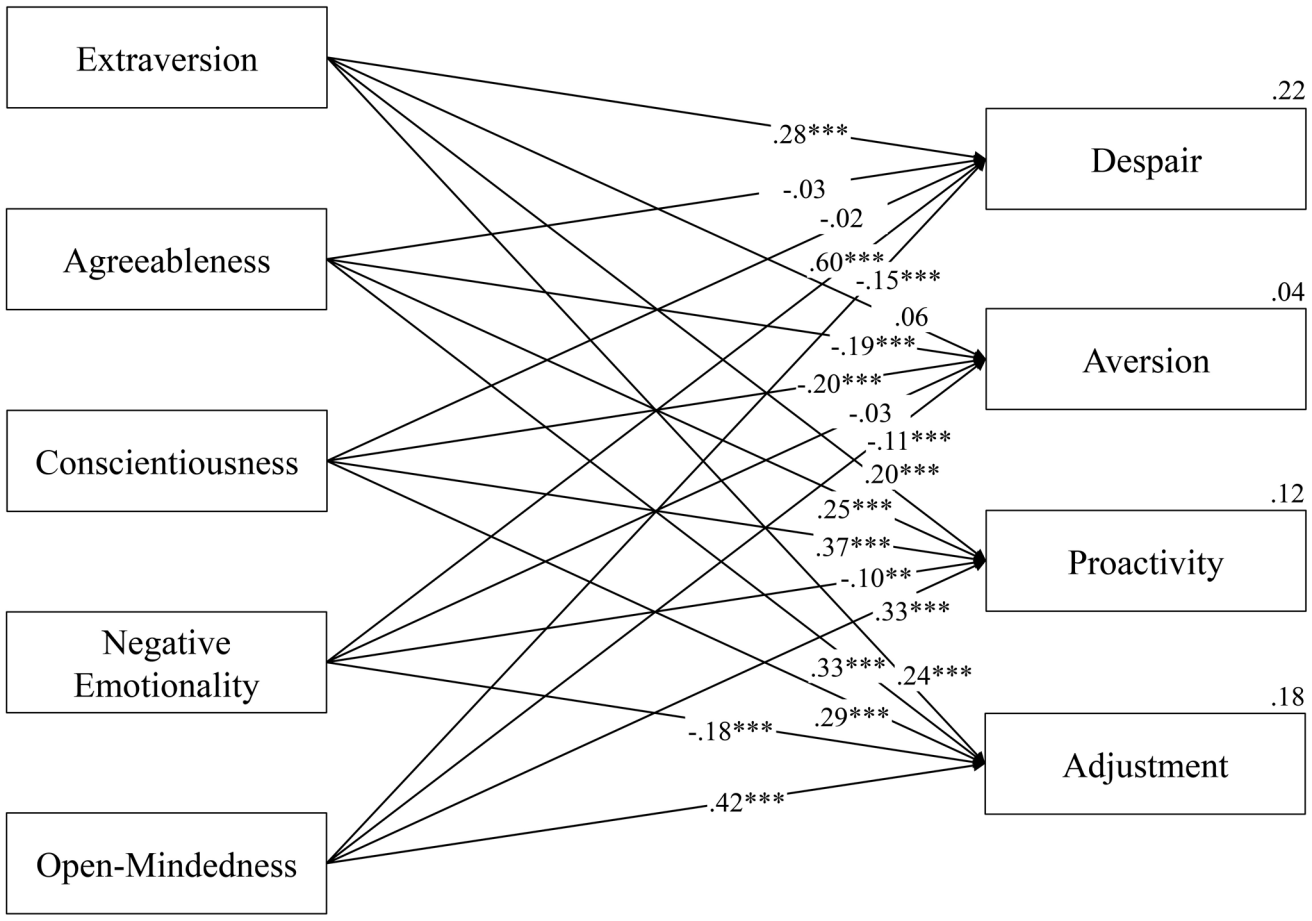
Path analysis for the relations between the five factors of the BFI-2-XS and the four dimensions of the R-PCS. The digits represent standardized factor loadings. We reported explained variances next to each dependent variable. *p  <  0.05, **p  <  0.01, ***p  <  0.001.

The results of the PA are represented in Figure 2. Here we obtained a just-identified model, i.e., a model in which the degrees of freedom were tantamount to zero, resulting in a single, unique solution where the model accurately reproduces the data. Therefore, indices such as the Comparative Fit Index (CFI—compares the fit of the data to the hypothesized model), the Tucker-Lewis Index (TLI—compares the fit of the hypothesized model to a null model), the Goodness of Fit Index (GFI—measures the compatibility of the observed covariance matrix with the hypothesized model), and the Adjusted Goodness of Fit Index (AGFI—corrected version of GFI that accounts for the number of indicators per latent variable) were all found to be equal to 1.000; additionally, the Root-Mean-Square Error of Approximation (RMSEA) and the Standardized Root Mean Residual (SRMR), which evaluate the difference between the hypothesized model and a perfect model, and the discrepancy between the sample covariance matrix and the model covariance matrix, respectively, were also found to be equal to 0.000 (Hu and Bentler, 1999; Marsh et al., 2004; Kline, 2016; Cook and Forzani, 2020).

Despair was significatively and positively linked with Extraversion, *β* = 0.283, *p* < 0.001, and with Negative Emotionality, *β* = 0.600, *p* < 0.001. It was also significatively and negatively related to Open-Mindedness, *β* = −0.148, *p* < 0.001. For these three traits, our data fully supported Hypothesis 1. The links between Despair and, respectively, Agreeableness, *β* = −0.030, *p* = 0.299, and Conscientiousness, *β* = −0.022, *p* = 0.455, were not significant, but the negative direction was the one expected on the basis of Hypothesis 1. For Despair, the five traits explained on the whole the 22% of variance.

Aversion had significant negative links with Agreeableness, *β* = −0.194, *p* < 0.001, Conscientiousness, *β* = −0.200, *p* < 0.001, and Open-Mindedness, *β* = −0.108, *p* < 0.001, in line with Hypothesis 2. The relations with Extraversion, *β* = 0.057, *p* = 0.162, and Negative Emotionality, *β* = −0.027, *p* = 0.315, were not significant, but they were however in the expected direction. For this coping dimension the explained variance was 4%.

Proactivity was significantly related to each of the five traits, positively to Extraversion, *β* = 0.197, *p* < 0.001, Agreeableness, *β* = 0.247, *p* < 0.001, Conscientiousness, *β* = 0.367, *p* < 0.001, and Open-Mindedness, *β* = 0.332, *p* < 0.001, and negatively to Negative Emotionality, *β* = −0.100, *p* = 0.001. Explained variance was 12%. These findings confirmed Hypothesis 3.

Similarly, Adjustment was significantly linked with all the traits, positively with Extraversion, *β* = 0.238, *p* < 0.001, Agreeableness, *β* = 0.334, *p* < 0.001, Conscientiousness, *β* = 0.288, *p* < 0.001, and Open-Mindedness, *β* = 0.418, *p* < 0.001, and negatively with Negative Emotionality, *β* = −0.180, *p* < 0.001. Explained variance was 18%. Our results supported Hypothesis 4.

## 6. Discussion

Our first analyses enabled us to describe how our sample is characterized in terms of personality and COVID-19-related coping (Aim 1). The Italian university students involved in the study had higher scores in Conscientiousness, followed by Open-Mindedness, and then Agreeableness. These three traits were, in turn, higher than Extraversion and Negative Emotionality. Concerning coping strategies, they reacted more frequently using adaptive reactions (with Proactivity used more frequently than Adjustment) rather than maladaptive reactions (with Despair higher than Aversion).

Focusing on the relation between personality and coping (Aim 2), our findings revealed that Negative Emotionality can be considered as a risk factor, and that Agreeableness, Conscientiousness, and Open-Mindedness can be thought as protective factors. More interestingly, we also found that Extraversion can entail both a risk and a protective role for Italian university students after the outbreak of the COVID-19 pandemic.

On the one hand, our analysis showed that students’ Negative Emotionality was significantly and positively related with Despair, a pattern of coping strategies focused on helplessness and negative emotional reactions, spanning from panicking to the complete absence of hope. This result confirms a very high number of previous findings documenting that neurotic people are prone to distress and psychopathology, both in a variety of situations (Bogg and Roberts, 2013; Friedman and Kern, 2014; Strickhouser et al., 2017; Anglim et al., 2020) and also during the COVID-19 pandemic (Qian and Yahara, 2020; Kocjan et al., 2021; Kohút et al., 2021; Nikčević et al., 2021; Schmiedeberg and Thönnissen, 2021). This is also in line with COVID-19 data about stress and wellbeing of secondary school and university students (Staller et al., 2021; Iterbeke and De Witte, 2022; Quigley et al., 2022; Wang et al., 2022; Zolotareva et al., 2022). Moreover, the relation between students’ Negative Emotionality and Despair was very strong and it was higher compared to the relation with the other four traits, again supporting previous COVID-19-related findings (Kocjan et al., 2021; Bacon et al., 2022). Concerning Aversion, the link with Negative Emotionality was negative but not significant. The negative direction is in line with the hypothesis that neurotic people are particularly compliant with rules, based on previous COVID-19 research about adults (Chan et al., 2020; Fink et al., 2021; Peters et al., 2023). However, prior findings reported no significant or positive relations (Bogg and Milad, 2020; Kohút et al., 2021). Nevertheless, most of these studies focused on specific types of safety behaviors, such as social distancing, disinfecting, reducing mobility, etc. The absence of a significant relation in our data could be explained by the fact that the Aversion dimension lacked this specificity, rather focusing on oppositive behaviors towards any kind of protective measures proposed by the authorities. Concerning the two adaptive coping dimensions, Negative Emotionality was significantly and negatively linked with both of them. Even if neurotic people had demonstrated an active search for information during the pandemic (Kohút et al., 2021), their typical tendency to use maladaptive coping and low assertiveness (Costa and McCrae, 2006; Balzarotti et al., 2010;Bagherian and Mojambari, 2016; Sims, 2017) probably prevented students from adapting successfully during the outbreak of the pandemic. Therefore, our data confirmed that Negative Emotionality is a risk factor for Italian university students in the impact phase of a disaster such as the current pandemic, as it had been amply demonstrated previously for a variety of situations.

On the other hand, Agreeableness, Conscientiousness, and Open-Mindedness clearly revealed their protective role. In our database, they were generally significantly related with each of the four factors, negatively with Despair and Aversion (albeit with some exceptions, in which the direction of the links was in line with our hypotheses but the relations were not significant) and positively with Proactivity and Adjustment. Again, this is in line with both pre-pandemic literature and the COVID-19 studies. Concerning Despair, the negative links suggested an overall lower distress for agreeable, conscientious, and open-minded students. Even if the COVID-19 findings are not always consistent, they had suggested that these three traits were characterized by a good positive functioning in terms of wellbeing and mental health (Kocjan et al., 2021; Nikčević et al., 2021), and this had been in part documented also with secondary and university students (Audet et al., 2021; Staller et al., 2021; Iterbeke and De Witte, 2022; Wang et al., 2022; Zolotareva et al., 2022), confirming pre-pandemic data (Anglim et al., 2020). If we focus on Aversion, the tendency to care for others (Habashi et al., 2016; Sims, 2017), to respect rules (Barrick and Mount, 1991; Roberts et al., 2005), and to adapt smoothly to new situations (Schmutte and Ryff, 1997; LePine et al., 2000; McAdams, 2015) of people high in each of the three traits, respectively, could help explaining their low opposition towards protective measures. The same characteristics, together with the tendencies to be assertive (Bagherian and Mojambari, 2016; Sims, 2017) and to use reappraisal (Kohút et al., 2021), can justify the positive links between the three traits and the two adaptive coping dimensions.

Finally, our analysis showed that Extraversion can be both a risk and a protective factor in the impact phase of a disaster. Research had generally supported the potentialities of extraverted people as being characterized by subjective wellbeing (Li et al., 2015; Anglim et al., 2020), with this trait being protective for both wellbeing and mental health indicators also during the pandemic (Kocjan et al., 2021; Nikčević et al., 2021). However, for our sample of Italian university students there was a significant and positive relation between Extraversion and Despair, in line with some research involving adults during the COVID-19 pandemic (Kocjan et al., 2021; Kohút et al., 2021; Schmiedeberg and Thönnissen, 2021) and also university students (Anglim and Horwood, 2021; Wang et al., 2022; Weiß et al., 2022). Interestingly, similar findings did not emerge in a study with university students involved during the spring of 2021, for which there were positive relations between Extraversion and stress management (Zolotareva et al., 2022). Our result concerning Extraversion gives particular support to the relevance of the concepts of goodness-of-fit and person–situation interaction (Goldberg, 1993; Faulkner et al., 2004) when evaluating the advantages and disadvantages of people’s dispositions. In other terms, individuals’ characteristics are associated with a positive psychological functioning and adaptation whether they are matched in certain ways with specific characteristics of the situations. An ongoing question in personality research is the extent to which the positive or negative feelings of wellbeing experienced by extraverts are primarily determined by the person’s own psychological constitution, or by the external interactions they have with other people (Audet et al., 2021). The COVID-19 pandemic had an undoubtedly devastating impact on human communities across the world. Nevertheless, it did provide a natural laboratory for exploring this question about personality. In many parts of the world (Acaps, n.d.), lockdown conditions imposed by governments meant that face-to-face interactions between people were at the very least, seriously limited. Our results seem to reveal that, for the specific group of university students for which most of the usual face-to-face social interactions were suddenly and abruptly denied, deprivation of social contact had the worst effects for extraverted people, tipping the balance of such debate towards the importance of interindividual processes. Their subjective wellbeing was thus proved to be highly dependent from external factors (Goryńska et al., 2015), in line with their reluctance to follow recommendations (Fink et al., 2021; Krupić et al., 2021; Peters et al., 2023), especially when concerning social distance from others (Bacon et al., 2022). The positive (even if not significant) link between Extraversion and Aversion, indeed, supported their difficulties in complying with the rules imposed by the authorities to reduce the spreading of the virus. We could speculate that the non-significance of this link could be due, again, to the non-specific characterization of Aversion: in other terms, it is probable that we would have found a significant relation if such dimension had only referred to social distancing. As for the relation between Extraversion and the two adaptive coping dimensions, the data fully confirmed previous literature, showing that extravert students reacted also in adaptive ways, through active problem solving, looking for information and adjusting positively to the challenges of the situation, capitalizing on key strengths such as their assertiveness (Balzarotti et al., 2010) or reappraisal use (Bagherian and Mojambari, 2016; Sims, 2017).

Referring again to the frequency with which our sample was characterized by the Big Five traits and the different coping strategies documented examining our first aim, we could speculate that, overall, the pattern which emerges seems to be adaptive. Beyond endorsing more frequently adaptive rather than maladaptive strategies, our university students were also characterized by higher scores for those personality traits which confirmed their protective role, i.e., Agreeableness, Conscientiousness, and Open-Mindedness.

### 6.1. Limitations and future directions

The current study has a number of limitations. First, our sample was characterized by gender imbalance and included students from one country, i.e., Italy. Researchers should try to examine cross-cultural data (e.g., Raccanello et al., 2022a) to support the generalizability of their findings. Second, we used self-report instruments, limited by constraints related to social desirability or memory biases. However, they are still the best ways to access inner states and their use was one of the few available ways to conduct research during the lockdown periods. Third, the scale utilized to assess the Big Five traits was only 15 items long, and this could have impacted its reliability (Soto and John, 2017). However, this measure enabled to minimize the assessment time, an essential feature for a large-scale survey with a within-subject design, and this limitation is common to most of the studies conducted during the COVID-19 pandemic. Future studies could generalize our findings using longer instruments. Fourth, we assessed also the specific facet traits within each of the broader Big Five domains, but given the short nature of the instrument we could not study them. Further research analyzing different facets could help to better disentangle some ambiguous findings highlighted in the literature about the relation between the Big Five traits and coping during the pandemic. Fifth, we did not examine how trait profiles were associated with coping, which could be considered in future research to better describe risk and protective factors. For example, extraverted people who are particularly conscientious could have suffered more compared to those with lower Conscientiousness, given their strong sense of responsibility soliciting them to respect rules, including those about social distancing. Sixth, we utilized a cross-sectional design, which is correlational in nature. Nevertheless, the on-course analysis of the data that we gathered longitudinally could help to draw causal conclusions about the relations between the examined variables. Seventh, we measured personality traits using one specific model, assessing the Big Five factors; future research could investigate the role of personality traits also using other models, such as the HEXACO model, in the subsequent phases of the pandemic, or in relation to other disasters.

### 6.2. Conclusion

Our findings shed light on the role of the Big Five traits towards Italian university students’ coping at the outbreak of the COVID-19 pandemic. We found support for the risk role of Negative Emotionality and the protective role of Agreeableness, Conscientiousness, and Open-Mindedness. However, in line with the person-situation perspective, this study revealed that Extraversion had both a risk and a protective role.

Knowledge on the nature of the association between personality traits and maladaptive or adaptive reactions when facing a completely unexpected traumatic event can be at the basis of the development of disaster preparedness and prevention actions (United Nations Office for Disaster Risk Reduction, UNDRR, n.d.). Supporting students to increase their awareness about which reactions are more probably associated with their personality, could be a way to improve their capacity to be better equipped to contrast the endorsement of maladaptive ones. In other terms, it could help to improve their resilience towards current or future disasters.

## Data availability statement

The raw data supporting the conclusions of this article will be made available by the authors, without undue reservation.

## Ethics statement

The studies involving human participants were reviewed and approved by Ethical Committee of the Department of Human Sciences of the University of Verona (protocol n. 118846/2020). The patients/participants provided their written informed consent to participate in this study.

## Author contributions

RB, DR, and GV contributed to conception and design of the study and organized the database. RB performed the statistical analysis. RB and DR wrote the first draft of the manuscript. GV wrote sections of the manuscript. All authors contributed to the article and approved the submitted version.

## Conflict of interest

The authors declare that the research was conducted in the absence of any commercial or financial relationships that could be construed as a potential conflict of interest.

## Publisher’s note

All claims expressed in this article are solely those of the authors and do not necessarily represent those of their affiliated organizations, or those of the publisher, the editors and the reviewers. Any product that may be evaluated in this article, or claim that may be made by its manufacturer, is not guaranteed or endorsed by the publisher.
